# Objections to assisted dying within institutions: systemic solutions for rapprochement

**DOI:** 10.1186/s12910-023-00981-2

**Published:** 2023-11-16

**Authors:** Carmelle Peisah, Adrianna Sheppard, Kelvin CY Leung

**Affiliations:** 1 Discipline of Psychiatry and Mental Health, Faculty of Medicine, University of New South Wales, Sydney, NSW Australia; 2 Specialty of Psychiatry, Faculty of Medicine and Health, University of Sydney, Sydney, Australia; 3 Capacity Australia, Sydney, Australia; 4 Research and Education Network, Westmead Hospital, Sydney, NSW 2145 Australia

**Keywords:** Voluntary assisted dying, Medical assistance dying, Conscientious objection

## Abstract

In this Matters Arising article, we outline how the recent article “The impact on patients of objections by institutions to assisted dying: a qualitative study of family caregivers’ perceptions” (White et al., 2023 Mar 13;24(1):22) informed Voluntary Assisted Dying (VAD) implementation in our large Australian public health setting, where objections do not emanate from, but within, the institution. In reporting the harms to patients and caregivers created by institutional objection, White et al. provide an evidenced-based road map for potential potholes or risks associated with VAD implementation. We discuss the complexities emerging from the diverse views of health professionals and the ethical tensions arising from such, especially within certain specialties, and how we developed systemic strategies that support patients, caregivers and staff alike. We highlighted the need to shift from “Do you support VAD?“ to “How can we support you as healthcare professionals to integrate VAD into your practice, in a way that complies with the legislation, meets the needs of patients and caregivers, and feels safe and does not compromise your moral stance?“

We welcomed publication of the recent article “The impact on patients of objections by institutions to assisted dying: a qualitative study of family caregivers’ perceptions” by White et al., [1] as an evidenced-based road map for potential potholes in the implementation of voluntary assisted dying (VAD)(also known as medical assistance in dying). We consider White et al’s., [1] findings extremely salient as healthcare environments across the globe adopt VAD. We recommend they be heeded particularly by those of us in the implementation phase, finding relevance for this paper beyond objections by institutions, to objections within institutions. As such, White et al., [1] informs an important element of risk management in VAD implementation. We write this Matters Arising Article to demonstrate how we used the White et al [1] paper to inform systemic strategies to deal with the predicted risks, including the ethical tensions, associated with VAD implementation in a public health setting. We do so from the perspective of a senior human rights psychiatrist (CP) and two psychiatry registrars (AS and KL) working within a medical Professional Support Unit in an education network within a large Australian Local Health District.

White et al., [1] found that institutional objections impacted assessment for eligibility, access to, use and administration of VAD medication, and was associated with delays and barriers to VAD choice, including dichotomising choice between VAD or palliative care, not both. Most relevantly, objections can be overt and explicit, as expressed through religious institutional objection, or, most relevant to public health settings entrusted with statutory obligations for implementation, expressed covertly or obliquely by staff.

In highlighting the impact of institutional objection to VAD on patient care, White et al [1] provided powerful examples of how systemic factors can influence the success of VAD implementation. In capturing the patient and caregiver voice, White et al [1] drew attention to the practical and emotional experience of patients accessing VAD, fundamental to which are interactions with staff, including not only those directly providing VAD but also those responding to patient queries.

Distinct from the rationale for institutional objection described by White et al [1], objection by healthcare professionals is motivated by a broad range of moral, religious, professional and political commitments, motivated by concerns for patients, self and the medical profession [2]. In turn, the varied nature and strength of these objections shapes the way health professionals envisage speaking to patients about VAD, leading to classification as dissuasive or passive non-referrers, facilitators or negotiators [3]. Notwithstanding this, such anticipated responses remain purely speculative, as ultimately the legislation outlines the obligations of objectors, albeit variably so depending on the jurisdiction. Moreover, while some doctors support VAD legislation in theory, even lower numbers are actually willing to participate in VAD [4], a distinction critical in recruiting an actual VAD workforce.

Other determinants of support or objection amongst healthcare professionals are discipline and specialty. While approximately 73% of healthcare workers overall support VAD legislation, only 51% of medical specialists do so, with lowest support coming from palliative care and geriatric specialists [4, 5]. VAD is often seen as antithetical to the professional *raison d’être* of the palliative care professional, as evidenced by statements from international palliative care groups excluding VAD provision from palliative care practice [6–8]. Notwithstanding this, there is an ongoing debate concerning VAD within palliative care specialists and national societies of palliative care, and not all eschew VAD. For example, Dutch and Belgian palliative care institutions have allowed VAD within their walls, and Canadian palliative care is involved in MAID provision, at least in some regions [9–13].

Often the discourse around conscientious objection around VAD is focused solely on dangers and harms, with suggested strategies bringing objectors into line and silencing them. Instead, we purposefully modelled a stance of “critical neutrality” [14], noting Gilbert’s institutional “duty to be neutral” [15]. The Royal Australian College of Physicians notes that this stance encourages “*respectful and reflective dialogue with patients, families and colleagues who might have differing views…. an approach that avoids imposing one’s own values on another person in favour of helping them find their way to a conclusion consistent with their own set of values”*[16, p9]. With VAD implementation, we suggest that systems move away from binary, dichotomised views of individuals, specialities or professional groups as advocates or objectors [5], and rather acknowledge the variation and complexity of health care professionals’ perspectives. This is consistent with the statutory requirements in our legislation but common to most VAD legislation, which notes: “all persons, including health practitioners, have the right to be shown respect for their culture, religion, beliefs, values and personal characteristics.” [17] This is essential given that staff objectors and advocates alike are equally vulnerable to being “named and shamed’ for their VAD stance [5], depending on the prevailing zeitgeist of the healthcare institution. Understanding moral injury and the need for job control and psychological safety of staff is integral to VAD implementation in healthcare institutions [6]. Moreover, we speculate that supporting staff in managing their VAD stance will also prevent passive-aggressive acting out of anger or anxiety about the process.

With this in mind, we adopted the following systemic strategies within our institution:

(i) We tested the “affect” (emotional tone) of the system, and raised awareness of this within the system, normalising and giving permission for all affective responses creating greater psychological safety. We observed a range of responses amongst staff from anxiety and fear (i.e. about what they would be “made to do”), confusion and uncertainty (e.g. about the legislation, and staff roles), anger (e.g. about VAD being foisted upon them) and shame for beliefs;
(ii) We named and reconciled the systemic split working towards rapprochement. We normalised and gave permission for the variation of VAD stances amongst staff, while acknowledging that we have “a job to do”, legislation to comply with, and patients to care for;
(iii) We modelled understanding and respect for ethical tensions created by VAD within certain specialities(e.g. geriatrics and palliative care), while asserting the right of all patients to access palliative care and VAD. This mitigated the risk identified by White et al [1] namely forcing patients into an either/or VAD or palliative care choice;
(iv) We extended White et al’s “reasonable accommodation” [1, p10] stance to all stakeholders to promote systemic cohesiveness and unity. Utilising strategies adopted elsewhere to facilitate a pragmatic stance amongst objectors, working with the legislation to the extent compatible with their own views – “if for no other reason than to protect the therapeutic alliance” with the patient [18, p374]. We provided education about how to manage personal objection(5) while remaining compliant with the legislation and optimising patient care, outlining how to respectfully deal with patient enquiries with information and referral options to VAD navigators and/or providers;
(v) We identified and named the process of undue influence, whereby some staff imposed their strongly-held views on others, particularly more junior staff, often nursing staff. We articulated the dangers of and set boundaries around this practice;
(vi) Acknowledging the complexity of VAD, we introduced peer support for VAD practice amongst health professionals.

In Table 1, we outline how we adopted these strategies.

**Table 1:** Working towards rapprochement between disparate ethical stances on Assisted Dying in health systems

Strategy	The how: what did we actually do?
Testing the “affect” of the system	We directly sought staff feedback about their feelings about VAD* (i.e. not by generic institutional surveys)
Naming and reconciling the systemic split	We emphasised and gave permission for the variety of VAD stances in all clinician forums.
Modelled understanding and respect for ethical tensions created by VAD	We modelled understanding and respect for conscientious objection while emphasising the essential integration of palliative care, both with leaders and across all clinician forums.
A stance of reasonable accommodation compliant with the legislation	Education about managing personal objection started with leaders first, and was subsequently delivered across the organisation. We disseminated the White et al [1] paper.
Identifying and addressing undue influence	Openly discussed power imbalances within the organisation. The importance of giving voice to quieter members of the system repeatedly emphasised in multidisciplinary clinician forums
Peer Support Groups	This was focused on clinicians actively involved in VAD.

Key *VAD = Voluntary assisted dying

Unaddressed, adverse systemic responses to VAD may cause harm, as illustrated by White et al [1], as can systemic fracture and splitting. Using the principle of forewarned is forearmed, we used the White et al. paper and others’ experience to anticipate systemic responses to VAD and implement systemic strategies to tackle these complexities. Notwithstanding the myriad of challenges faced, we tried to walk the talk in actualising the rights of all persons to be shown respect for their culture, religion and values. White et al [1] call for better regulation and policy to address this problem. We also call for adjunctive systemic strategies that make it safe for all stakeholders regardless of beliefs, while raising awareness about the harm of such when acted out with patients and families.

## Data Availability

N/A.

## Abbreviations

VAD: Voluntary Assisted Dying
N/A: Not applicable
